# Dr. Maclean, in Answer to Dr. Drake, on Digitalis

**Published:** 1800-02

**Authors:** L. Maclean


### *To the Editors of the Medical and Phyſical Journal.*

Gentlemen,

In the Medical and Phyſical Journal for November laſt, is inſerted a very ingenious Eſſay, on the Digitalis Purpurea, from the pen of Dr. Drake. This Eſſay ſeems to have originated from ſome haſty remarks introduced by me to public notice, through the ſame channel, in Auguſt laſt. A principal motive with with me, in bringing forward theſe obſervations, was to draw the attention of the medical world more immediately to this highly important ſubject, and to ſtimulate others to its farther proſecution; more eſpecially as I ſaw, with infinite concern, men holding forth expectations from it in the cure of conſumption, which I was confident, from my own experience, would never be realized. But while I derive no ſmall degree of ſatisfaction from ſeeing this part of my object in a fair train of being fully accompliſhed, I cannot be ſuppoſed to view with indifference an attempt to overturn, by general aſſertion, without argument or proof, what has been laid down as facts by me; and certain opinions attributed to me, which it never entered my mind to advance, much leſs to ſupport.

This Eſſay, therefore, demands ſome notice from me; and it is incumbent upon me to prove, that what I have advanced as facts, are ſo in reality; leſt I ſhould incur the charge of deceiving, while profeſſing to inſtruct thoſe who are yet to learn the general properties of the Fox-glove. Neceſſary, however, as this may, in every point of view, be, the publication of ſome obſervations I have lately drawn out, muſt unavoidably be delayed ſome time longer. They have been extended to a length that precludes their inſertion in your valuable Journal, for which they were intended, ſhould they be deemed worthy of a place in it.

You will therefore have the goodneſs to announce my intention of publiſhing, ſome time in the Spring, "An Eſſay on the general Properties of the Fox-glove; in which, the extent of its virtues in conſumption, and various other diſeaſes; its doſes, mode of action, preparation, and exhibition, are pointed out; together with an analyſis and refutation of Dr. Darwin's Theory, which ſuppoſes hectic fever to be the conſequence of the abſorption of aerated, or oxygenated, matter or pus; to which are ſubjoined, three caſes of inſanity ſucceſsfully treated by it."

The facts alluded to above, regard chiefly the growth and quality of the plant of my own garden, and the ſtrength of the tinctures of which I have given formulæ.

I can aſſure Dr. Drake, he is compleatly deceived in what he has ventured with ſo much confidence to aſſert, reſpecting the ſituation of my houſe, the ſoil of my garden, and the powers of the plant reared in it. If the ſituation of my houſe be "certainly low and damp," as the Doctor has affirmed, it has hitherto eluded my own and my family's obſervation. The fox-glove has for ſome years flouriſhed with the greateſt luxuriance in my garden. In ſome places the habitudes of the plant have been moſt attentively conſulted, and gratified, by means of an artificial ſoil and favourable expoſure; in others, however, it thrives thrives among many other ornamental flowers, where the ſoil is naturally light, dry, and ſandy, with little care in the culture; and I have aſcertained, beyond the poſſibility of doubt, by comparative experiments made, varied, and multiplied, in numberleſs inſtances, that the ſtrength and virtues of the leaf thus reared, are not inferior to thoſe of the natural growth. I can, moreover, venture to aſſure thoſe who may wiſh to have the plant within their immediate reach, which gives the moſt decided advantages, that they will find no difficulty in its cultivation.

With regard to the tinctures of which I have given formulæ, I can adduce the teſtimony of almoſt every medical practitioner with whom I am in conſtant habits of acting, in proof of what I have advanced on a former occaſion. I have never been able, except in one inſtance, to exceed the quantity of 90 drops in a day; and I generally found from 60 to 75 drops fully ſufficient to affect powerfully the ſtrongeſt conſtitutions. In the exception mentioned, three draughts with 32 drops of the tincture of fox-glove, and 15 of laudanum in each, were taken in one day, and a fourth on the following morning; but the whole frame was ſo much under its influence, that, although the medicine was diſcontinued for a day prior to my viſit, I was ſeriouſly alarmed for the ſafety of my patient. The ſame perſon is now unable to continue, for any length of time, 60 drops in a day. When I perceive that Dr. Drake has exhibited 120 and 100* drops in twenty-four hours, of the tincture, which he calls ſtronger than mine, I cannot but expreſs my ſurpriſe at his aſſertion. It may be proper however to obſerve, that, although the colour of the tincture of the dried leaves, made according to the directions given by me, be dark, Dr. Darwin's, when firſt made, is darker, and, when immediately evaporated, while warm, leaves a greater quantity of reſiduum. But, when removed to the cool temperature of the apothecary's ſhelf, it depoſits a conſiderable portion of ſediment, and conſequently becomes proportionably weaker; and, I have reaſon to believe, does not ultimately retain ſo much of the active parts of the leaf in ſolution as that which is digeſted for a week with only half the quantity. Even this laſt depoſits ſome ſediment. Hence the grounds on which the proportions I ventured to ſubmit to the conſideration of the learned College were founded.

On the ſolid baſis of experiment too, I have aſcertained that every effect which the tincture is capable of producing, may be equally expected from the powder alſo; and, as I have already obſerved,

* Medical and Phyſical Journal, Vol. II. p. 267, et ſeq.

observed, the only advantage the former has over the latter is, that the dose can with more facility be regulated. Yet, although the tincture was the only preparation used by Dr. D. in every case he has as yet brought before the public, (and we are unavoidably compelled to infer that he has published the full result of his experience to the date of his last communication) he has affirmed,* "that the wished for effect in consumption can seldom be produced by the powder." I suspect he has himself, before this time, discovered, "that the wished for effect" will seldom be produced, "even by the tincture, in genuine hereditary scrophulous consumption," although he has with confidence affirmed, that it would, "in general, cure" this hitherto almost uniformly fatal disease. By giving this a place in your next Number, you will much oblige,

Your obedient servant,

L. MACLEAN.

N. B. I beg, in this place, to acknowlege my obligations to Dr. Moffman, for his very candid and liberal interpretation of some hints I took the liberty of suggesting for his consideration in your Journal, Vol. II. p. 177.

